# Polymorphisms at Candidate Genes for Fat Content and Fatty Acids Composition: Effects on Sheep Milk Production and Fatty Acid Profile Using Two Dietary Supplementations

**DOI:** 10.3390/ani13152533

**Published:** 2023-08-06

**Authors:** Serena Tumino, Matteo Bognanno, Giorgio Chessari, Marco Tolone, Salvatore Bordonaro, Fabrizio Mangano, Donata Marletta, Marcella Avondo

**Affiliations:** 1 Dipartimento di Agricoltura, Alimentazione e Ambiente, University of Catania, 95123 Catania, Italy; serena.tumino@unict.it (S.T.); giorgio.chessari@phd.unict.it (G.C.); fabrizio.mangano@unict.it (F.M.); mavondo@unict.it (M.A.); 2 Dipartimento di Scienze e Tecnologie Agro-Forestali e Ambientali, University of Reggio Calabria, 89100 Reggio Calabria, Italy; matteo.bognanno@unirc.it; 3 Dipartimento di Scienze Agrarie, Alimentari e Forestali, University of Palermo, 90128 Palermo, Italy; marco.tolone@unipa.it

**Keywords:** ovine milk, *DGAT1* 5′UTR polymorphism, *SCD* 5′UTR polymorphism, carob pulp, barley grain, fatty acid profile, lipid health indices

## Abstract

**Simple Summary:**

The fat component of sheep milk exhibits significant variability. While nutrition is widely acknowledged as the main factor affecting fat yield and fatty acid profile in ruminants’ milk, genetic factors also contribute to this variation at breed and individual levels. This study aimed to examine the impact of genotypes and feed integration on milk quality in Valle del Belìce sheep. To achieve this aim, two polymorphic lipogenic genes (*DGAT1* and *SCD*) and the impact of two dietary supplementations (carob pulp and barley grain) were investigated. Carob pulp, a local agro-industrial by-product rich in carbohydrates and tannins, was selected due to its potential to positively affect ruminant metabolism, suggesting a viable and sustainable alternative for ruminant feeding from economic and environmental perspectives. The genotype at the *DGAT1* locus significantly affected both the milk urea content and milk fatty acid composition. The carob pulp supplementation, compared to barley, increased the fat percentage but worsened the milk fatty acid profile in terms of healthy properties. The interaction between genotypes and diet was not evident.

**Abstract:**

The nutritional value of sheep’s milk and its derivatives is influenced by the lipid fraction, which is affected by diet and genetics. This study aimed to explore the genetic variations in the *DGAT1* and *SCD* genes and assessed the impact of the *DGAT1* genotype on milk quality in Valle del Belìce sheep, considering diet supplementation with carob pulp and barley grain. Among the potentially polymorphic sites, only *DGAT1* g.127 C > A and *SCD* g.87 C > A showed variability. The *DGAT1* genotype did not significantly impact milk yield and composition, except for higher urea content in the CA genotypes than in the CC ones. Carob pulp increased the milk fat content compared to barley grain. Genetic variation in *DGAT1* was associated with changes in the milk fatty acid profile; specifically, the CA genotype exhibited higher levels of short-chain fatty acids and lower levels of polyunsaturated fatty acids compared to the CC genotype. Carob pulp supplementation increased saturated fatty acids and reduced unsaturated fractions, leading to milk with higher atherogenic and thrombogenic indices. No significant interaction was found between genotype and diet. This study provides insights into the genetic and dietary factors influencing sheep’s milk composition. Further research is needed to understand the impact of these genetic variations on milk production and composition, as well as to determine optimal levels of carob pulp for improving fat percentage and promoting sustainable sheep breeding practices.

## 1. Introduction

Sheep’s milk is characterized by high protein and fat content, which are definitely exploited in the cheesemaking process. In Mediterranean countries, ovine milk is either processed on farms or sold to the industry for transformation into niche, ethnic, or typical dairy products [1]. Ovine high-quality products, like Pecorino cheese in Italy, are included in the list of ingredients of the Mediterranean diet [2] that is recognized to have a positive impact on the cognitive functioning of healthy older adults [3] and to be effective in reducing the risk of cardiovascular diseases and overall mortality [4,5]. The nutritional interest in ovine milk primarily revolves around its fat composition. The FA profile of milk, particularly the content of essential PUFAs, linoleic acid (LA, C18:2 *n*-6) and α-linolenic acid (ALA, C18:3 *n*-3), plays a significant role in determining its nutritional value [6,7]. Sheep milk naturally contains high levels of short-chain saturated FAs (SCFAs), long-chain FAs (LCFAs), and polyunsaturated FAs (PUFAs) [8]. The nutritional quality of milk lipids can be enhanced by reducing the fraction of saturated long fatty acids in favor of the unsaturated ones through appropriate dietary strategies and, at least potentially, by genetic improvement. Indeed, mammary gland lipid metabolism involves numerous genes [9], and over the last few decades, an increasing number of genetic polymorphisms, mainly single nucleotide polymorphisms (SNPs) within several candidate genes affecting fat content and fatty acid composition, have been described in many species, including sheep [10,11].

*DGAT1* and *SCD* are two particularly interesting candidate genes for improving the nutritional quality of milk fats. *DGAT1* encodes for Diacylglycerol O-acyltransferase 1, an enzyme that plays a key role in the final step of triacylglycerol biosynthesis, whereas *SCD* encodes for the Stearoyl-CoA desaturase, an endoplasmic reticulum (ER) enzyme that catalyzes the biosynthesis of monounsaturated fatty acids (MUFAs) from newly synthesized or dietary-derived saturated fatty acids. Some polymorphisms have been identified in both ovine *DGAT1* and *SCD* genes, some of which have been studied for their association with milk and meat production traits in different breeds. Both genes contain SNPs in the 5′UTR promoter region, which are potential candidates for playing a crucial role in regulating gene expression [12,13]. Additionally, the regulation of these genes can also be influenced by environmental factors and feeding composition [14,15,16].

In traditional Mediterranean breeding systems, which heavily rely on grazing resources, lactating ewes often receive a dietary supplement in the stable. The quality and quantity of the supplementation contribute to correct, improve, and balance the diet of lactating ewes. Supplementation with barley grain represents a common practice in the Mediterranean dairy sheep system, although the high feed cost makes it quite challenging. In this view, carob pulp, a locally available and cost-effective agro-industrial by-product, represents a more sustainable and economically viable alternative to barley [17]. Carob pulp, derived from the processing of carob fruits or pods from the Carob tree (*Ceratonia siliqua* L.) (Fabaceae), is widely cultivated in the Mediterranean area and is commonly used in sheep diet supplementation [18,19]. Its high content in soluble sugars and tannins can influence microbial functions in the rumen [20].

The current research is part of an investigation on the effects of some genotypes, different feed supplements and the interaction between genotypes and nutrition on the production and nutritional qualities of sheep’s milk. The first results have been reported recently [21]. In this study, we examined the genetic polymorphisms of two candidate genes that could impact the fat content and fatty acid composition in Valle del Belìce ewes. Additionally, through a feeding trial, we assessed the impact of the total replacement of barley grain, one of the most common supplements for grazing sheep, with carob pulp, on milk production and fatty acid composition in grazing ewes with known genotypes at the *DGAT1* and *SCD* loci.

## 2. Materials and Methods

### 2.1. Ethical Approval

All of the animals were managed according to the guidelines of the Animal Ethics Committee (O.P.B.A.) of the University of Catania (prot. No.158467).

### 2.2. Genetic Characterization

Genetic characterization was conducted on 77 Valle del Belìce lactating ewes. Genomic DNA was extracted from milk somatic cells using a modified salting-out method [22]. DNA quality and concentration were quantified with a NanoDrop 1000 spectrophotometer (NanoDrop Technologies, Wilmington, DE, USA). Genotypes at *DGAT1* and *SCD* loci were analyzed by direct Sanger sequencing. The *DGAT1* variants, g.127 C > A and g.358 C > A, at the 5′UTR region and exon 1, respectively, were investigated by sequencing an amplicon of 360 bp obtained according to Scatà et al. [13]. Three SNPs, localized in the promoter and 5′UTR regions of the *SCD* gene (g.87 C > A, g.257 G > A, and g.379 A > T) were detected by the direct sequencing of an amplicon of 527 bp which included the partial sequence of the promoter, the complete sequence of the 5′UTR, exon 1, and the partial sequence of the intron [12]. Table 1 provides comprehensive information regarding the individual SNPs and the protocols used in the study. PCR products (360 bp and 527 bp) were purified by Exo-SAP digestion followed by dideoxy sequencing with a BigDye™ Terminator v3.1 Cycle Sequencing Kit (Thermo Fisher Scientific Inc., Waltham, MA, USA). Sequencing reactions were performed bidirectionally on an Applied Biosystems 3130 genetic analyzer (Applied Biosystem, Foster City, CA, USA). All samples were sequenced. The sequences were aligned and compared with the respective reference sequences (GenBank Acc. Num. EU178818, JX944472, JX944473, JX944475) using MEGA X v.11 [23].

### 2.3. Animals and Feeding Management

Based on the observed polymorphism, as outlined in detail within the ‘Results’ section, it was found that the *DGAT1* g.127 C > A SNP was the only locus that exhibited a balanced distribution of two genotypes (CC and CA). Consequently, a total of 38 ewes with uniform characteristics in terms of lactation days (DIM 107 ± 14 days) and milk yield (820 ± 257 g/day) were chosen based on their genotypes at the *DGAT1* g.127 C > A locus and subsequently divided into two blocks (referred to as ‘a’ and ‘b’). The distribution of *DGAT1* g.127 C > A genotypes within each block was as follows:

Block a: 14 ewes with the g.127 CC genotype and 6 ewes with the g.127 CA genotype.

Block b: 12 ewes with the g.127 CC genotype and 6 ewes with the g.127 CA genotype.

All of the selected sheep were homozygous at the locus *SCD* (g.87 CC). The selected ewes were arranged in an experimental design consisting of two simultaneous change-over designs for two *DGAT1* genotypes (CC and CA), with one block assigned to each diet, the barley diet and the carob diet. Here are the details of the two diets:

- Barley diet: Net energy for lactation 1100 kcal/kg DM, crude protein 55 g/Kg DM, WSC (water-soluble carbohydrates) 485 g/Kg DM, NDF (neutral detergent fiber) 322 g/Kg DM, total polyphenols 17.9 mg/g DM, and tannins 12.5 mg/g DM.
- Carob diet: Net energy for lactation 1700 kcal/kg DM, crude protein 129 g/Kg DM, WSC 52 g/Kg, NDF 251 g/Kg DM, total polyphenols 2.8 mg/g DM, and tannins 1.5 mg/g DM.

The arrangement of treatments followed a 2 × 2 factorial design, considering both the two *DG*AT1 genotypes and the two diets. Each ewe grazed for 8 h on a mixed pasture and individually received the dietary supplementation in two equal portions during the two daily milkings in the barn. Specifically, the supplementation included 250 g/d of barley whole grain (group Barley) or 250 g/d of carob pulp (Group Carob). The experimental trial lasted 40 days, taking place from 21 March to 30 April. The main experiment was preceded by a 12-day pre-experimental period during which all of the animals grazed on the same pasture of the main experiment for 8 h each day. During this period, they also received a supplement consisting of 125 g of barley and 125 g of carob, which was distributed during their two daily milkings. The overall experimental design followed a change-over design, divided into two experimental periods, each lasting 20 days. The animals gradually adapted to the experimental diets in the first 12 days of each period. In the remaining 8 days of each period, the animals received the experimental, scheduled diets, and data were collected. Table 2 reports the detailed experimental plan.

### 2.4. Pasture Chemical Composition

At the beginning of each experimental period, pasture features, biomass, and herbage height were measured by cutting herbage on 8 plots of 1 × 1 m randomly distributed over the pasture. For each experimental period, six pasture samples were analyzed for their gross composition, phenolic compounds, total tannins, and fatty acids profile, as described by Tumino et al. [21].

### 2.5. Milk Ceasurements and Analysis

Individual milk production and samples were collected from both morning and afternoon milkings during the pre-experimental period, which lasted for 12 days, and at the end of each experimental period, which lasted for 8 days (at days 7 and 8). The milk samples were divided into two aliquots for analysis. One aliquot was analyzed immediately after collection for fat, lactose, protein, casein, and urea content (Milkoscan FT1 supplied by FOSS, Hillerød, Denmark). The second aliquot was treated with sodium azide 99% to prevent coagulation and stored at –30 °C for the analysis of milk fatty acid profile, as described by Tumino et al. [21]. Atherogenic (AI) and thrombogenic (TI) indices were estimated using the following formulas [26]:

(1) $$\frac{AI=\left[\left(C12:0+4\left(C14:0\right)+C16:0\right)\right]}{⅀UFA}$$

(2) $$\frac{TI=(C14:0+C16:0+C18:0)}{\left[\left(0.5\times ⅀MUFA\right)+\left(0.5\times ⅀n-6PUFA\right)+\left(3\times ⅀n-3PUFA\right)+\left(n-3/n-6\right)\right]}$$

### 2.6. Statistical Analysis

The frequencies of genotypes and alleles, as well as the Hardy–Weinberg equilibrium for individual SNPs, were calculated using POPGENE software version 1.32 [27].

Individual data on milk yield and composition, as well as fatty acids, were analyzed using the repeated measures GLM procedure (SPSS for Windows, Inc., Chicago, IL, USA). The statistical model incorporated the following factors: diet, *DGAT1* genotype, blocks (a and b), periods, and *DGAT1* genotype x diet. Milk production and gross composition recorded during the pre-experimental period were utilized as covariates. Covariates with no significant effects were excluded from the model. The LSD test was used to determine significant differences between the groups.

## 3. Results

### 3.1. Genetic Characterization

In the examined flock, a sample of 77 Valle del Belìce lactating ewes exhibited a moderate polymorphism at the 5′UTR and promoter regions of *DGAT1* and *SCD* loci (Table 3). The sequencing analysis of the 360 bp-long amplicon in the *DGAT1* 5′UTR did not reveal any new polymorphic site. Only g.127 C > A SNP and two genotypes were detected, with the C allele and the CC genotype being the most frequent, whereas the AA genotype was not identified in this Valle del Belìce sheep sample. The g.358 C > A SNP described by Dervishi et al. [24] in the Assaf breed appeared monomorphic in our Valle del Belìce sample, with only the C allele detected. Similarly, the *SCD* 5′UTR and promoter regions were analyzed through direct sequencing to identify at least three potential SNP sites: the g.87 C > A, g.257 G > A, and g.379 A > T. No new SNPs were discovered for the analyzed loci, and only the g.87 C > A locus was found to be polymorphic (Table 3); three genotypes were observed, with CC being the most prevalent. The haplotypes CGA and AGA were also observed, with CGA being the predominant one (87.3%). No significant deviation from Hardy–Weinberg equilibrium was observed for the loci analyzed.

Due to the uneven distribution of the genotypes at the two tested loci, only 38 ewes with CC (26) and CA (12) genotypes at the *DGAT1* locus, all homozygotes at the *SCD* (g.87 CC) locus, were included in the experimental groups for the subsequent feeding trial.

### 3.2. Effect of Genotype and Dietary Integration on Milk Production and Fatty Acid Composition

The genotype (g.127 CC vs. CA) of *DGAT1* 5′UTR did significantly impact milk yield and gross composition, except for urea content which was significantly higher in the CA ewes (Table 4). The integration with carob pulp significantly increased the milk fat content compared to supplementation with barley grain. Valle del Belìce ewes that received barley supplementation produced slightly more milk; due to this not significant difference, during the trial, they produced daily significantly more protein (58.08 vs. 45.02 g/d; *p* = 0.002), casein (46.02 vs. 36 g/d; *p*= 0.003), and lactose (42.46 vs. 33.74 g/d; *p* = 0.024).

The fatty acids are presented in Table 5 and Table 6. The genotype significantly influenced certain fatty acids: *DGAT1* g.127 CA ewes’ milk was richer in butyric (C4), caproic (C6) and total short-chain saturated fatty acids (SCFAs), whereas it had lower levels of lauric (C12), heptadecanoic (C17), iso-heptadecanoic acid (C17:0 iso), cis-9-heptadecenoic acid (C17:1 c9), linoleic (C18:2 c9c12), and PUFAs (Table 5). The diet strongly affected the fatty acid profile; the inclusion of carob pulp enriched the milk with myristic (C14), palmitic (C16), C17, myristoleic (C14:1 c9), cis-11-octadecenoic acid (C18:1 c11), C18:2 c9c12, total SFA, and total MCFA, whereas it reduced C4, C6, C17:0 iso, trans-10-octadecenoic acid (18:1 t10), vaccenic (C18:1 t11), conjugated linoleic acid (C18:2 c9t11), total SCFAs, total MUFAs, total PUFAs, and total trans fatty acids. Furthermore, carob pulp increased the milk atherogenic and thrombogenic indices (Table 6). No significant genotype x diet interaction was evident in our experimental condition, either on the milk fatty acid profiles or on the nutritional indices.

## 4. Discussion

*DGAT1* and *SCD* are candidate genes associated with fat production and fatty acid profile. Numerous studies have investigated their genetic polymorphisms in various cattle breeds [28,29,30,31], identifying SNPs in coding regions associated with milk and meat fat content, fatty acid profile, and other productive traits, including fertility [32,33,34,35,36,37,38]. However, studies on minor species such as goats, buffalo, and sheep are limited [39,40,41]. In sheep research, there is low variability at these loci [12,24], and most studies focus on polymorphisms in coding regions. Regarding the *DGAT1* gene, the SNPs in exons 10, 16, and 17 have been extensively investigated for their association with meat traits in sheep [41,42,43,44,45,46]. In a study by Dervishi et al. [24] on the Assaf breed, the entire coding region of the ovine *DGAT1* gene was sequenced in 50 sheep from five Spanish breeds to identify polymorphisms and their potential association with milk traits. Four SNPs were identified: one in exon 1 (g.358 C > A), two in exon 17 (g.8522 C > T and g.8539 C > T), and one SNP in intron 10 (g.7457 C > A). The SNP g.8539 C > T, previously observed in Asian and Italian breeds, appeared to be partially associated with their productive specialization (mainly meat and wool) and the presence of adipose depots in the fat tail. Regarding the *SCD* gene, studies have primarily focused on examining the variability of exons 3 and 8 and their impact on meat fatty acid profiles and quality traits, including lamb meat tenderness [47,48].

In our study, we sequenced two amplicons, measuring 360 bp and 527 bp, to investigate the genetic variation in the 5′UTR of *DGAT1* and *SCD* genes, respectively. We observed a moderate level of polymorphism in 77 Valle del Belìce ewes. Out of the five SNPs potentially present in the 5′UTR and promoter regions of *DGAT1* and *SCD* genes, only two polymorphic sites were identified (*DGAT1* g.127 C > A and *SCD* g.87 C > A), while the remaining loci were monomorphic (*DGAT1* g.358 CC, *SCD* g.257 GG, and *SCD* g.379 AA), with no new genetic variations observed. Similar findings, with a few exceptions, have been detected in various sheep breeds. For instance, Dervishi et al. [24] reported that only two out of nine analyzed Spanish sheep breeds exhibited polymorphism at the *DGAT1* g.358 C > A locus, albeit at a low allele frequency. Furthermore, the SNP mutation *SCD* g.257 G > A was found to occur at a very low frequency in three Iranian breeds [25], while the *SCD* g.379A > T locus displayed good variability. It should be noted that the polymorphisms reported here in Valle del Belìce are mapped in non-coding but regulatory regions, in which mutations may directly or indirectly control transcription regulation and gene expression.

To contextualize our findings, we compared the observed genetic variability with the limited data available in the literature. The SNP in the *DGAT1* 5′UTR (g.127 C > A), within the core sequence of the Sp1 transcription factors binding site, was originally detected by Scatà et al. [13] in three Italian dairy breeds (Altamurana, Gentile di Puglia, and Sarda). The mutated allele A was found at low frequency in Altamurana (0.05) and Gentile di Puglia (0.02), which are dual- and triple-purpose breeds, defined by the authors as “high-fat” milk due to their milk composition. In contrast, the Sarda breed, a specialized dairy breed with high milk yield and medium-to-low-fat milk percentage, exhibited 19% of the frequency of the mutated A allele. Furthermore, the authors carried out an association study in Sarda sheep, revealing a significant negative association between *DGAT1* 5′UTR (g.127 C > A) SNP and milk fat and protein content, as well as a positive association with milk yield in this dairy breed. Considering the potential effects of g.127 C > A on milk production and quality, further investigation is desirable in other dairy sheep populations. The focus of our study is the Valle del Belìce breed, a Sicilian dairy breed that originated in the 20th century through crossbreeding between autochthonous Sicilian breeds and selectively chosen Sardinian breed rams [49]. It is known as one of the most productive Italian dairy breeds. The breed’s origin and its productive aptitude may explain the presence of the g.127 A allele in Valle del Belìce.

As previously mentioned, sequencing of the *SCD* 5′UTR region in the Valle del Belìce breed revealed only one polymorphic site (g.87 C > A). This SNP, located in the promoter region, was previously reported by Garcìa-Fernàndez et al. [12] as g.31 C > A according to GenBank Acc. N. FJ513370, along with other SNPs in the gene’s first exon and first intron, in a large and heterogeneous sample of Mediterranean breeds. The frequency of the mutated allele A observed in Valle del Belìce (0.14) agrees with the findings reported by Aali et al. [25] in 458 fat-tailed and thin-tailed Iranian sheep. In a highly diverse sample including eight French, Spanish, and Egyptian sheep breeds with different morphology (fat tail vs. thin tail) and productive specialization (milk vs. meat), the rare allele A in the promoter region (referred to as g.31 C > A) showed a moderate frequency of almost 22% across the entire sample [12,50]. The distribution of this variant among different breeds was uneven and suggested a potential correlation of variant A with the degree of specialization of the dairy breeds, that is, a positive association with milk yield and a negative one with milk fat content. In fact, the A allele displayed a very low frequency in a large sample of meat breeds from Chile [51] while it exhibited higher frequencies (0.32 and 0.35) in Rasa Aragonesa [52] and in a sample of 50 Slovak dual-purpose sheep (0.37) [53]. This polymorphism has never been investigated in any Italian breed.

Overall, our findings suggest the existence of discrete genetic variability in the 5′UTR and the promoter regions of the *DGAT1* and *SCD* genes within the Valle del Belìce dairy breed, underscoring the need for further efforts to collect phenotypes associated with these polymorphisms.

The primary objective of this study was to examine the impact of genotypes at specific loci within the putative transcription regulatory regions of the *DGAT1* and *SCD* genes, as well as the influence of two different feed integration approaches commonly used in our traditional semi-extensive system, and their interaction with milk yield, gross composition, and milk fatty acid profile in Valle del Belìce sheep. However, due to the limited variability observed in the considered population and the imbalanced distribution of genotypes found at *SCD* g.87 C > A locus, it was not possible to form feeding groups with similar numbers of animals. Consequently, the feeding study, designed as a 2 × 2 changing scheme, was conducted using exclusively ewes’ homozygotes at the *SCD* (g.87 CC) that differed only in their *DGAT1* g.127 genotypes (CC and CA). Under our experimental conditions, the genotype did not significantly influence milk yield and fat composition in contrast with results reported in the Sarda breed [13]. Gross composition was not affected except for the urea content, which was higher in the CA sheep (*p* = 0.023; Table 3). It is worth noting that no studies are currently available on the impact of this SNP on sheep milk urea content. Although it is difficult to hypothesize a biological link of the polymorphism under study on the level of urea in milk, Van Gastelen et al. [54] demonstrated the influence of the DGAT1 genotype on nitrogen (N) efficiency in Holstein–Friesian cows. Indeed, in ruminants, the N levels found in milk and blood are closely connected to the efficiency of N utilization by rumen microbes, which facilitate the conversion of ammonia into microbial protein. Any surplus of ammonia is absorbed across the rumen wall, subsequently undergoing a transformation into urea and being excreted through urine and milk [55].

The dietary supplementation with carob pulp increased the fat percentage (*p* < 0.001) with no other effect on milk yield and composition. The replacement of barley with carob pulp may influence rumen degradation kinetics and rumen fermentation efficiency due to its composition being rich in sugars and varying tannin levels. As a consequence, a reduction in the urea content of milk would have been expected, which instead, although showing a trend in this direction, did not reach statistical significance. The literature reports few studies on the effect of carob pulp on milk traits, while more studies focus on growing animals [56,57]. In general, the results, regarding milk, growth, and meat quality traits are rather contradictory. No effects were found on ewes milk [58] and growing lambs’ traits [59], while contrasting effects were reported in other experimental conditions [60], probably as an effect of the level of carob inclusion. Often, when testing diets with different levels of carob inclusion, a threshold value is highlighted, below which the carob can have a positive or neutral effect, whereas beyond this threshold, a decline is usually observed. However, this threshold value is not universally applicable. Further studies are necessary to determine the effect and the optimal levels of carob pulp inclusion in the diet of lactating ewes, in order to promote the use of this inexpensive agri-food by-product readily available in the Mediterranean area, in the diet of small ruminants for the economic and environmental sustainability of breeding.

Diacylglycerol acyltransferase enzymes (DGAT-1 and DGAT-2) catalyze the final step of triacylglycerol (TAG) formation. The main effect of *DGAT1* polymorphisms is the variation observed in milk yield, as well as milk fat and protein percentage. These effects have been reported across different species, including cows, buffaloes, goats, and sheep [61]. Moreover, studies have reported the influence of *DGAT1* polymorphisms on fatty acid composition in both cow milk [15,28,62] and sheep milk [24]. In the Assaf breed, Dervishi et al. [24] observed significant effects of the SNP g8539C > T on the C4, C16:1 c9, and n6/n3 content. It is known that the DGAT1 enzyme is responsible for the esterification of fatty acids on diacylglycerol in the sn3 position, and the acids occupying this position are mainly represented by SCFAs and the UFAs [63]. Consistent with the function of DGAT1, our experimental conditions showed that the SCFAs and the PUFAs were the categories of acids most affected by the polymorphism at 5′UTR *DGAT1*. Specifically, the presence of the allele A in heterozygous ewes with g.127 CA was associated with milk richer in SCFAs and lower levels of 12:0, 17:0, and PUFAs. Overall, under our conditions, the presence of the mutated allele g.127 A slightly worsened the nutritional quality of the milk in terms of fatty acid profile and thrombogenic index.

Comparing dietary supplementation with carob pulp to barley grain, it was found that carob pulp led to a deterioration in the milk fatty acid profile. It increased the level of total SFA and MCFA (14:0 and 16:0, respectively) whilst reducing the level of total SCFAs, as well as the unsaturated fractions (MUFAs and PUFAs) and 18:2 c9t11 (CLA), leading to a worsening of health lipid nutritional indices. It would have been expected that the use of a supplement rich in tannins, such as carob, compared to barley, could have improved the acid composition of the milk fat. This expectation is based on the inhibitory effect of tannins on ruminal biohydrogenation, as demonstrated in other studies [64]. This inhibition could potentially lead to a reduction in SFA and an increase in PUFAs in the milk of the group fed with carob. However, as previously hypothesized [21,65], the effects produced by tannins depend not only on their concentration and structure but also on the chemical–physical structure of the feeds and their interaction with the pasture.

Even if the use of carob pulp did not improve the composition of the lipid fraction, the nutritional index values reported here with both supplements are quite good, probably due to grazing feeding, which is widely recognized for its contribution to improving the nutraceutical quality of small ruminant products. In fact, the values obtained in this study are comparable with the range values reported for sheep’s milk (AI 1.42–3.39 and TI 1.00–2.72) [66] and with those recorded in milk produced on mountain pastures at over 2200 m (AI 2.35 and TI 2.48) [67].

These two nutritional indices are commonly used in literature to assess the effects of dietary fat on human health; they consider the diverse impact of individual fatty acids, specifically their potential to influence the occurrence of pathological events, such as atheroma and thrombi formation [66]. This information is important because sheep milk’s lipid fraction is found in processed products such as cheese, which are part of the Mediterranean diet. While no medical organization has yet provided the recommended values for the AI and TI indices, the consumption of food with low health lipid indexes is recommended to maintain good physical and mental health [66].

## 5. Conclusions

Under our experimental conditions, genetic variation at *DGAT1* g.127 C > A locus slightly affected the milk composition, showing an association with the urea content in milk and the fatty acid profile in a semi-extensive feeding system. Feed supplementation had a more pronounced influence on milk and fat composition, as well as nutritional quality. The milk produced under these experimental conditions had good AI and TI nutritional indices, likely due to the extensive use of natural grazing.

Further research is needed to gather more phenotypic data associated with these polymorphisms in Valle del Belìce sheep in order to gain a better understanding of their impact on milk production and composition. Additionally, the optimal level of the inclusion of carob pulp in the diet of lactating sheep needs to be defined, considering its effect on fat percentage and its potential for economic and environmental sustainability in Mediterranean sheep breeding.

## Figures and Tables

**Table 1:** Gene Bank accession numbers, polymorphic sites, and primers used for genotyping.

Gene	Acc. Num.	Region	SNP	Amino Acid Substitution	Primers Sequence (5′-3′)	Amplicon Size	Reference
*DGAT1*	EU178818	5′UTR	g.127 C > A	-	GGAACTACGCTTCCCAGGAC ACGTCTCCGTCCTTGTCTGT	360	[13]
		Exon 1	g.358 C > A	p.Asp53Glu			[24]
*SCD*	JX944472	Promoter	g.87 C > A	-	AAATTCCCTTCGGCCAATGAC TCTCACCTCCTCTTGCAGCAA	527	
	JX944475	Promoter	g.257 G > A	-			[25]
	JX944473	5′UTR	g.379 A > T	-			

**Table 2:** Experimental plan details.

Periods	Days	Block a (20 Ewes)	Block b (18 Ewes)
Pre-experimental	12 days	125 g barley + 125 g carob pulp + 8 h on a mixed pasture	125 g barley + 125 g carob pulp + 8 h on a mixed pasture
Period 1			
Diet adaptation	12 days	Progressive adaptation to the experimental diets	
Data collection	8 days	250 g barley + 8 h on a mixed pasture	250 g carob pulp + 8 h on a mixed pasture
Period 2			
Diet adaptation	12 days	Progressive adaptation to the experimental diets	
Data collection	8 days	250 g carob pulp + 8 h on a mixed pasture	250 g barley + 8 h on a mixed pasture

**Table 3:** Genotype and allele frequencies at the *DGAT1* and *SCD* loci in Valle del Belìce sheep.

SNP	Genotypes Frequency		Allele Frequency		N	χ^2^	*p*
*DGAT1* g.127 C > A	CC CA AA	0.75 0.25 -	C A	0.88 0.12	58 19 -	1.52	0.216
*DGAT1* g.358 C > A	CC CA AA	1.00 0 0	C A	1.00 0	77 - -	-	-
*SCD* g.87 C > A	CC CA AA	0.74 0.25 0.01	C A	0.86 0.14	57 19 1	0.17	0.676
*SCD* g.257 G > A	GG GA AA	1.00 0 0	G A	1.00 0	77 - -	-	-
*SCD* g.379 A > T	AA AT TT	1.00 0 0	A T	1.00 0	77 - -	-	-

**Table 4:** Milk yield and gross composition.

	*DGAT1* (G)		Diet (D)		Significance			SEM
	CA	CC	Carob	Barley	G	D	G × D	
Milk yield g/d	840.0	836.1	784.1	892.0	0.953	0.103	0.897	25.7
Fat %	6.50	6.40	6.98	5.92	0.712	<0.001	0.588	0.16
Protein %	6.16	6.43	6.17	6.42	0.107	0.131	0.793	0.06
Lactose %	4.49	4.64	4.55	4.59	0.171	0.661	0.680	0.04
Urea mg/dL	30.8	26.4	26.9	30.4	0.023	0.068	0.501	0.99
Casein %	4.90	5.14	4.94	5.09	0.119	0.314	0.789	0.06

SEM: standard error of the mean.

**Table 5:** Milk fatty acid composition (% total fatty acids).

	*DGAT1* (G)		Diet (D)		Significance			SEM
	CA	CC	Carob	Barley	G	D	G × D	
4:0	2.68	2.33	2.31	2.71	0.006	0.002	0.180	0.07
6:0	2.42	2.25	2.24	2.43	0.043	0.025	0.371	0.04
8:0	2.47	2.41	2.39	2.48	0.532	0.314	0.635	0.05
10:0	7.28	7.54	7.50	7.32	0.322	0.497	0.896	0.15
12:0	3.92	4.24	4.20	3.97	0.037	0.152	0.964	0.08
14:0	10.4	10.6	10.9	10.1	0.271	0.001	0.848	0.11
14:1 c9	0.21	0.21	0.24	0.18	0.890	0.030	0.245	0.01
16:0	22.6	21.7	22.8	21.5	0.207	0.045	0.395	0.24
17:0 iso	0.34	0.37	0.34	0.37	0.001	0.002	0.364	0.01
17:0	0.75	0.78	0.79	0.74	0.023	<0.001	0.749	0.08
17:1 c9	0.22	0.25	0.24	0.23	0.034	0.185	0.798	0.01
18:0	9.21	8.63	8.87	8.97	0.082	0.759	0.788	0.17
18:1 t10	2.97	3.20	2.68	3.49	0.325	0.001	0.862	0.16
18:1 t11	0.43	0.45	0.41	0.46	0.303	0.010	0.070	0.01
18:1 c9	15.7	15.6	15.8	15.5	0.827	0.518	0.435	0.20
18:1 c11	0.66	0.65	0.73	0.59	0.826	0.001	0.985	0.04
18:2 c9c12	1.65	1.78	1.80	1.63	0.004	<0001	0.272	0.03
18:2 c9t11	1.39	1.62	1.35	1.67	0.120	0.029	0.568	0.08
18:3 α	1.73	1.87	1.73	1.88	0.082	0.057	0.926	0.04

SEM: standard error of the mean.

**Table 6:** Milk fatty acid classes and nutritional index (% total fatty acids).

	*DGAT1* (G)		Diet (D)		Significance			SEM
	CA	CC	Carob	Barley	G	D	G × D	
SFA	64.2	62.7	64.4	62.4	0.125	0.037	0.691	0.53
SCFA	7.57	7.00	6.94	7.62	0.029	0.010	0.276	0.14
MCFA	21.6	22.4	22.6	21.4	0.141	0.041	0.980	0.31
MUFA	24.5	24.9	24.2	25.2	0.434	0.043	0.728	0.26
OBCFA	4.62	4.78	4.69	4.71	0.060	0.830	0.718	0.04
*Trans* FA	4.95	5.20	4.54	5.61	0.435	0.001	0.881	0.20
PUFA	4.82	5.28	4.88	5.22	0.004	0.029	0.507	0.08
AI	2.38	2.29	2.47	2.20	0.304	0.005	0.847	0.04
TI	2.20	2.06	2.24	2.02	0.027	0.001	0.773	0.03

SFA: saturated fatty acids; SCFA: short-chain fatty acids; MCFA: medium-chain fatty acid; MUFA: monounsaturated fatty acids; OBCFA: odd and branched-chain fatty acids; PUFA: polyunsaturated fatty acids; AI: atherogenic index; TI: thrombogenic index; SEM: standard error of the mean.

## Data Availability

Data supporting the findings of this study are available from the corresponding authors (S.B and D.M.) on request.
